# A case of digital vasculitis in anti-synthetase syndrome (Anti-OJ subtype)

**DOI:** 10.1186/s41927-025-00484-0

**Published:** 2025-03-27

**Authors:** Deniz AK, Richard J. Stratton

**Affiliations:** 1 Rheumatology, Royal Free Hospital London, London, UK; 2 Division of Medicine, University College London, London, UK

**Keywords:** Anti-synthetase syndrome, Anti-OJ, Digital vasculitis, Vasculopathy, Organising pneumonia, Interstitial lung disease

## Abstract

Anti-synthetase syndrome is a rare autoimmune disorder characterised by the presence of autoantibodies against aminoacyl transfer RNA synthetases. We report a unique case of a 54-year-old woman with anti-OJ anti-synthetase syndrome, characterised by the atypical occurrence of digital vasculitis in conjunction with the classic manifestations of anti-synthetase syndrome. Our patient presented with digital vasculitis affecting the right third and fourth fingers, rapidly evolving interstitial lung disease of the organising pneumonia subtype, sub-clinical myositis, arthritis and mechanic’s hands. Notably, she had no prior history of Raynaud’s phenomenon. Serological tests revealed positive anti-OJ antibodies and weakly positive anti-MI2 antibodies. Our patient’s condition was managed with intravenous methylprednisolone then after stepped down to prednisolone and mycophenolate mofetil with successful therapeutic response.

Current literature primarily highlights Raynaud’s phenomenon and vasculopathy-related ischemia, whether occlusive or non-occlusive in anti-synthetase syndrome. This case study identifies digital vasculitis as a distinctive complication of anti-synthetase syndrome, anti-OJ subtype. It emphasises the importance of recognising vascular complications, including vasculitis, even when classic signs like Raynaud’s phenomenon are absent. Further research is crucial to fully understand the range of vascular manifestations associated with anti-synthetase syndrome.

## Background

Anti-synthetase syndrome is a rare autoimmune condition characterised by the production of antibodies against aminoacyl transfer RNA (tRNA) synthetases. It is recognised as one of the disorders under idiopathic inflammatory myopathies [1]. Presentations can include Interstitial lung disease (ILD), mechanic’s hands, non-erosive arthritis, myositis, Raynaud’s phenomenon, and unexplained fever [2]. To date, eight known antibodies have been identified, including anti Jo-1 (Histidyl-tRNA synthetase), anti PL-7 (anti-threonyl-tRNA synthetase), anti PL-12 (alanyl-tRNA synthetase), anti-EJ (glycyl tRNA-synthetase), anti-OJ (anti-isoleucyl-tRNA synthetase autoantibody), anti-KS (anti-asparaginyl-tRNA synthetase autoantibodies), anti-Zo (anti-phenylalanyl tRNA synthetase), and anti-HA/YRS (tyrosyl transfer-RNA (tRNA) synthetase) [1]. Anti-Jo-1 is the most common, while anti-OJ is among the less frequently observed antibodies [1]. In the literature concerning patients with anti-OJ antibodies, it has been observed that they typically either exhibit isolated ILD or, when myositis is present, it tends to manifest as a severe form [3]. Our patient displayed a more diverse and unique range of clinical features. We present the first case of a patient with anti-OJ antibodies with digital vasculitis alongside known features of anti-synthetase syndrome such as rapidly evolving ILD, arthritis, mechanic’s hand and sub-clinical myositis with her raised Creatinine Kinase (CK) levels.

## Case presentation

We present a case of a 54-year-old woman who received a new diagnosis of anti-OJ anti-synthetase antibody syndrome, characterised by rapidly progressive ILD - organising pneumonia subtype, arthritis, mechanic’s hands, and digital vasculitis.

**Fig. 1:**
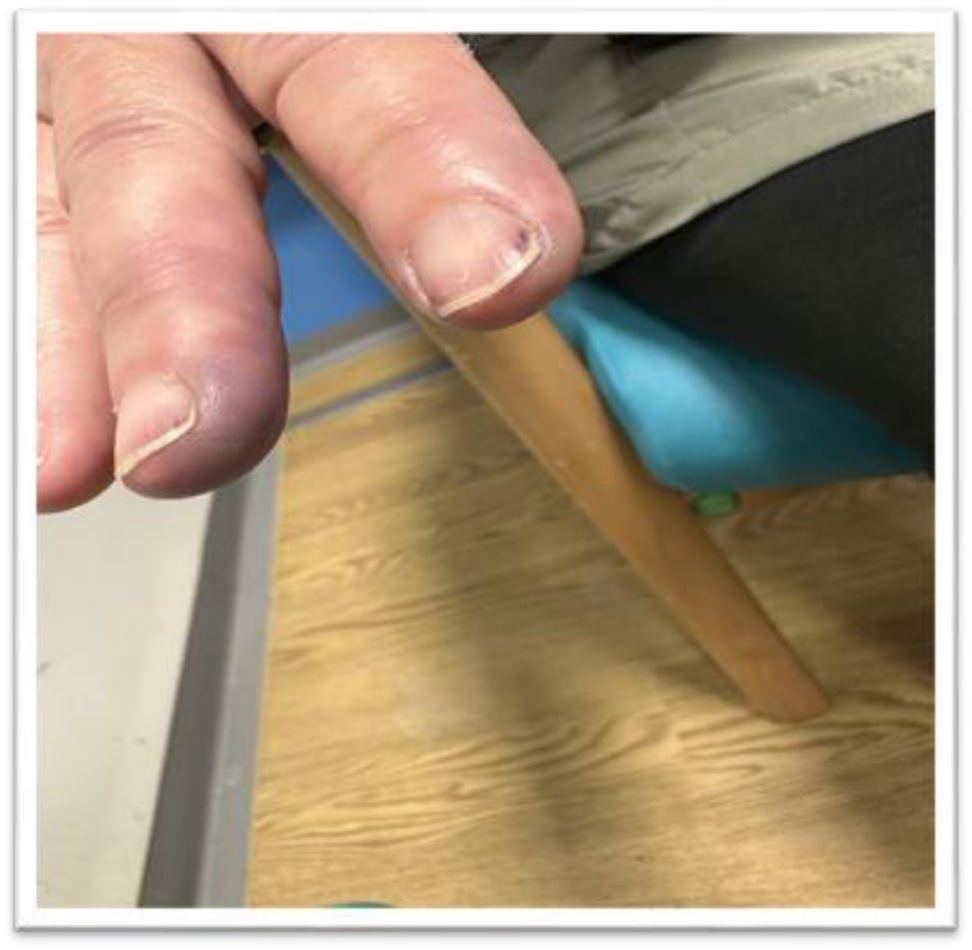
Imaging of the digital vasculitis of the 3rd and 4th fingers. Splinter haemorrhages could also be seen

Our patient presented to our hospital on August 23, 2024, with fixed bluish, dusky appearances in the third and fourth fingers of her right hand plus splinter haemorrhages, consistent with digital vasculitis (Fig. 1). These first started to appear two weeks prior. Her known past medical history included gastro-oesophageal reflux disease, hypercholesteremia, uterine fibroids, and reactive arthritis during her pregnancy.

Prior to this admission with digital vasculitis, over a six-month period, her primary complaints included progressively worsening breathlessness on exertion and palpitations, particularly noticeable when climbing stairs. In terms of her lungs, she also described difficulty taking deep breaths, a dry cough and a “feeling of rawness” in her chest. Further review of her systems revealed additional issues. She reported morning stiffness and puffiness in her finger and wrist joints, as well as knee pain during a recent holiday, which had since resolved. She also noted increased sensitivity and irritation in her eyes when using makeup or shampoo. Additionally, she experienced roughness in the skin of her hands. She had no family history of autoimmune conditions. She has never had Raynaud’s phenomenon and denies rashes.

She was referred to a cardiologist by her general practitioner. She did not have atrial fibrillation of note. Given her hypercholesterolemia, smoking 35 pack-year history along with an echocardiogram showing hypokinetic inferior, basal inferoseptum, and proximal inferolateral segments, the cardiologist referred her for a Computed Tomography (CT) Coronary Artery evaluation. This occurred on July 01, 2024, and revealed unexpected lung imaging findings of multiple incidental semisolid and solid nodules bilaterally mostly in a subpleural distribution.

**Fig. 2:**
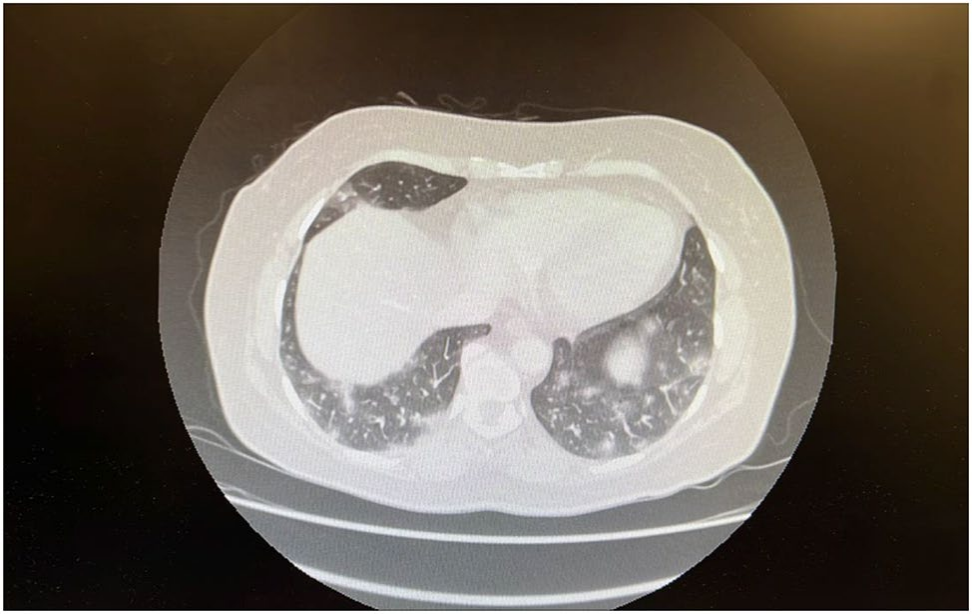
Cross sectional image of CT thorax with contrast images showing ILD

Further imaging to characterise these nodules on July 15, 2024, via CT thorax showed worsening of the previously found multifocal bilateral semisolid pulmonary nodules within a predominantly lower zone distribution, with possible early cavitation (Fig. 2).

The lung multi-disciplinary team were involved, raising the differential diagnosis of organising pneumonia versus atypical infection. The respiratory team arranged serological tests, lung function tests, and a bronchoscopy due to the imaging findings.

Serological tests in July included a myositis panel, which showed positive OJ antibodies and weakly positive Mi2b. CK levels were not measured at that time. The bronchoalveolar lavage results from a bronchoscopy performed on August 8, 2024, were sent for cytology analysis which showed no malignant cells, as well as for microbiology analysis indicating no fungal growth, no acid-fast bacilli and no significant microbial growth. Her lung function tests on August 07, 2024, indicated a borderline restriction pattern with a reduced force vital capacity of 77% and a preserved FEV1/FVC ratio of 79.5% consistent with ILD.

Returning to her admission with digital vasculitis affecting her right third and fourth fingers on August 23, 2024. Her CK level was 1424. Infective endocarditis as a differential was ruled out during her admission with no vegetations shown on her transthoracic echo on August 27, 2024. She had normal renal function tests with an estimated glomerular filtration rate of > 90 and her urine dipstick were negative for blood and protein. Cryoglobulins were not detected, there were negative anti-neutrophilic cytoplasmic autoantibodies, negative anti-nuclear antibodies, negative double stranded DNA antibodies, negative extractable nuclear antigen antibodies, negative rheumatoid factor, negative cyclic citrullinated peptide antibodies, negative anti-streptolysin O titre, negative syphilis serology, no abnormalities with serum electrophoresis and no paraproteins and normal complement levels.

Most importantly the doppler ultrasound performed on admission August 23, 2024, of our patient’s right limb revealed normal appearance of the brachial, radial, and ulnar arteries with triphasic waveforms. Normal appearance of the brachial, ulnar, radial, and median cubital veins with no evidence of thrombus. The proper digital arteries where visualized are patent with multiphasic waveforms. Clinically she has a normal vascular examination with palpable equal bilateral strong equal pulses felt across the radial and femoral arteries.

A CT Positron Emission Tomography (PET) scan completed on August 30, 2024, during her inpatient stay demonstrated marked further radiological progression of bilateral pulmonary findings with heterogeneous mild to moderate metabolic activity and three foci of hypermetabolic ground-glass nodular foci in the lower lobes. It was stated this would favour a systemic inflammatory process. No malignancy was shown.

An electrodiagnostic study performed on August 29, 2024, during her admission concluded with no evidence of active myositis noted, no evidence of mononeuritis multiplex but showed mild bilateral carpal tunnel syndrome. Wrist splints were provided. The bilateral carpal tunnel syndrome is likely secondary to wrist synovitis, consistent with the inflammatory arthritis component of anti-synthetase syndrome.

She was discharged with 10 mg of nifedipine and scheduled for outpatient follow-up with the diagnosis of anti-synthetase syndrome in mind. There was no improvement to her fixed dusky 3rd and 4th fingers in the community with nifedipine.

A cardiac magnetic resonance imaging completed as an outpatient on September 04, 2024, was consistent with autoimmune disease, mentioning potential late gadolinium enhancement in the right ventricle trabeculae which is non-ischemic. This can be a non-specific finding in some autoimmune diseases.

**Fig. 3:**
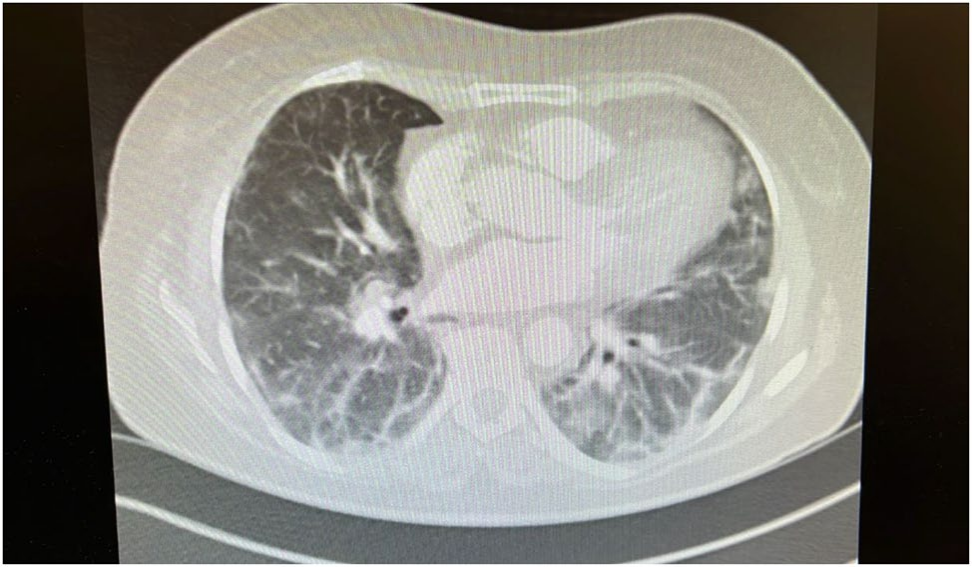
Cross-sectional image of CT Pulmonary Angiogram showing ILD that has progressed

She was later readmitted on September 15, 2024, after her general practitioner referred for dyspnoea with oxygen saturations of 82% with exertion. Her CK levels had increased to 2200 from 1424. The subsequent imaging of a CT Pulmonary Angiogram on September 17, 2024 was quoted to show no evidence of acute pulmonary embolus but an organising pneumonia ILD, seen to be progressing on sequential imaging over the last 2 months, likely related to the underlying autoimmune disease with a ground-glass/inflammatory component with a degree of mild fluctuation since August in keeping with ongoing exacerbation of her underlying ILD (Fig. 3).

The impression from the respiratory team clinically was that this is rapid progression of anti-synthetase organising pneumonia with limited evidence of superadded infection. Procalcitonin levels were also 0.03. She received three doses of 1 g intravenous methylprednisolone, which was subsequently stepped down to oral 10 mg prednisolone.

Regarding her follow up, she has been seen in our Rheumatology clinic and originally prescribed 500 mg of Mycophenolate Mofetil in liquid form, taken twice daily and has been escalated to 1000 mg twice daily. Her prednisolone has now been reduced to 5 mg on alternative days.

Clinically her digital vasculitis on the fingers have progressively improved with now normal appearances. There has been resolution of her mechanic hands and inflammatory joint pain, as well as improved breathlessness. Her CK levels have normalised to 49 from a peak of 2200.

Her repeat lung function on November 13, 2024, showed an improved forced vital capacity to 92.9% and FEC1/FVC ratio of 82.5%. Her repeat CT chest on February 11, 2025, compared with the CT pulmonary Angiogram on September 17, 2024, shows there has been almost complete interval resolution of the multifocal basal dominant pulmonary abnormalities with no new findings.

## Discussion

Although Raynaud’s phenomenon and digital ischemia are recognised in anti-synthetase syndrome, digital vasculitis remains an emerging complication.

Studies that demonstrate the above include Suma et al. who identified Raynaud’s phenomenon and myositis as predictors for the development of severe digital ischemia in patients with anti-synthetase syndrome [4]. Additionally, Yoshida et al. reported that 7% of patients with anti-synthetase antibodies experienced digital ischemia, often associated with systemic sclerosis-like phenotypes [5].

Several case reports have further elucidated the spectrum of vascular complications in anti-synthetase syndrome. Chan et al. described the first case of digital ischemia secondary to occlusive vasculopathy in a patient with anti-PL7/Anti-SSA 52kD antibodies [6]. They postulated that the underlying pathophysiology might involve microcirculatory arteritis, drawing parallels with a case reported by Disdier et al., where a patient with anti-Jo1 antibodies presented with digital ischemia [7].

The vascular involvement in anti-synthetase syndrome is not limited to the digits. Laugharne et al. reported a case of lower limb ischemia in a patient with anti-Jo1 antibodies, with their suggestion that vasculitis associated with autoimmune myositis could be the underlying mechanism [8]. Notably, there are emerging reports of patients presenting without a history of Raynaud’s phenomenon, suggesting a broader spectrum of vascular involvement in this syndrome. Abi Doumeth et al. For example, described a case of an anti-PL12 positive patient presenting with digital ischemia without prior Raynaud’s phenomenon, who also highlighted the possibility of diverse microangiopathic phenotypes in anti-synthetase syndrome [9].

Chan et al., Laugharne et al. And Suma et al. Demonstrate cases of obstructive vasculopathy whilst our case like Abi Doumeth et al. Anti-PL12 positive case had imaging showing patent digital vessels that are not opacified [4, 6, 8].

Horiuchi et al. presented a case of severe phalanx necrosis secondary to Raynaud’s phenomenon in a patient with anti-OJ and anti-Ro-52 antibodies, speculating that both vasospasm and vasculitis could contribute to the pathogenesis [10]. Unfortunately, Horiuchi et al.’s case does not have imaging of the underling vasculature; thus, it is unclear if this is obstructive or un-obstructive in nature [10].

We speculate that the underlying mechanism of digital vasculitis in this case is an immune-mediated endothelial injury rather than a vasospastic or thrombotic process. Although a tissue biopsy was not feasible due to the lesion locations, several clinical features support an inflammatory vasculopathy. The splinter haemorrhages on the nails likely reflect immune complex deposition and small vessel vasculitis. The fixed, pale, and poorly perfused appearance of the 3rd and 4th digits suggests digital artery involvement, consistent with a medium vessel vasculitis. The absence of symptom intermittency or temperature sensitivity, along with lack of response to vasodilator therapy, argues against a vasospastic process, while patent vasculature on Doppler imaging rules out thrombosis. Complete resolution with immunosuppressive therapy using prednisolone and mycophenolate mofetil further supports an immune-mediated mechanism.

The weakly positive anti-Mi2 antibody finding in our patient is of uncertain clinical significance. The overall clinical picture (ILD, mechanic’s hands, arthritis, subclinical myositis with elevated CK) is more consistent with anti-synthetase syndrome than dermatomyositis. In our clinical experience, incidental weakly positive antibodies are not uncommon when using comprehensive myositis panels. The patient lacked characteristic dermatomyositis features such as heliotrope rash, Gottron’s papules, or proximal muscle weakness.

## Conclusion

Our case report presents a unique manifestation: the first documented instance of a patient with anti-OJ antibodies presenting with digital vasculitis. This presentation occurred alongside other well-recognised features of anti-synthetase syndrome, including rapidly evolving ILD, arthritis, mechanic’s hands, and sub-clinical myositis evidenced by elevated CK levels.

The rarity of digital vasculitis in the literature underscores the significance of our case as a unique complication of anti-synthetase syndrome. As research into anti-synthetase syndromes progresses, digital vasculitis may emerge as a recognised complication, distinct from the more commonly reported Raynaud’s phenomenon and vasculopathy related ischemia.

This case highlights the importance of considering vascular complications, including vasculitis, in patients with anti-synthetase syndrome, even in the absence of typical features of vasculopathy like Raynaud’s phenomenon. Further research is needed to elucidate the full spectrum of vascular manifestations in anti-synthetase syndrome.

## Data Availability

Data related to this case is stored in the patient’s electronic medical records and is available upon reasonable request from the corresponding author.

## Abbreviations

tRNA: Transfer RNA
ILD: Interstitial Lung Disease
Anti-OJ: Anti-isoleucyl-tRNA synthetase autoantibody
Anti-MI2: Anti-MI2 antibodies
Anti-Jo-1: Anti-histidyl-tRNA synthetase
Anti-PL7: Anti-threonyl-tRNA synthetase
Anti-PL12: Anti-alanyl-tRNA synthetase
Anti-EJ: Anti-glycyl-tRNA synthetase
Anti-KS: Anti-asparaginyl-tRNA synthetase
Anti-Zo: Anti-phenylalanyl-tRNA synthetase
Anti-HA/YRS: Anti-tyrosyl-tRNA synthetase
CK: Creatine Kinase
CT: Computed Tomography
PET: Positron Emission Tomography
FEV1: Forced Expiratory Volume in 1 s
FVC: Forced Vital Capacity
